# Identification and Verification of Potential Therapeutic Target Genes in Berberine-Treated Zucker Diabetic Fatty Rats through Bioinformatics Analysis

**DOI:** 10.1371/journal.pone.0166378

**Published:** 2016-11-15

**Authors:** Yang Sheng Wu, Yi-Tao Chen, Yu-Ting Bao, Zhe-Ming Li, Xiao-Jie Zhou, Jia-Na He, Shi-Jie Dai, Chang yu Li

**Affiliations:** 1 College of Pharmacy, Zhejiang Chinese Medical University, Hangzhou, Zhejiang, People's Republic of China; 2 College of Life Science, Zhejiang Chinese Medical University, Hangzhou, Zhejiang, People's Republic of China; Max Delbruck Centrum fur Molekulare Medizin Berlin Buch, GERMANY

## Abstract

**Background:**

Berberine is used to treat diabetes and dyslipidemia. However, the effect of berberine on specific diabetes treatment targets is unknown. In the current study, we investigated the effect of berberine on the random plasma glucose, glycated hemoglobin (HbA1C), AST, ALT, BUN and CREA levels of Zucker diabetic fatty (ZDF) rats, and we identified and verified the importance of potential therapeutic target genes to provide molecular information for further investigation of the mechanisms underlying the anti-diabetic effects of berberine.

**Methods:**

ZDF rats were randomly divided into control (Con), diabetic (DM) and berberine-treated (300 mg⋅kg^−1^, BBR) groups. After the ZDF rats were treated with BBR for 12 weeks, its effect on the random plasma glucose and HbA1C levels was evaluated. Aspartate aminotransferase (AST), alanine aminotransferase (ALT), blood urea nitrogen (BUN), CREA and OGTT were measured from blood, respectively. The levels of gene expression in liver samples were analyzed using an Agilent rat gene expression 4x44K microarray. The differentially expressed genes (DEGs) were screened as those with log2 (Con vs DM) ≥ 1 and log2 (BBR vs DM) ≥ 1 expression levels, which were the genes with up-regulated expression, and those with log2 (Con vs DM) ≤ -1 and log2 (BBR vs DM) ≤ -1 expression levels, which were the genes with down-regulated expression; the changes in gene expression were considered significant at *P*<0.05. The functions of the DEGs were determined using gene ontology (GO) and pathway analysis. Furthermore, a protein-protein interaction (PPI) network was constructed using STRING and Cytoscape software. The expression levels of the key node genes in the livers of the ZDF rats were also analyzed using qRT-PCR.

**Results:**

We found that 12 weeks of berberine treatment significantly decreased the random plasma glucose, HbA1C levels and improved glucose tolerance. There was a tendency for berberine to reduce AST, ALT, BUN except increase CREA levels. In the livers of the BBR group, we found 154 DEGs, including 91 genes with up-regulated expression and 63 genes with down-regulated expression. In addition, GO enrichment analysis showed significant enrichment of the DEGs in the following categories: metabolic process, localization, cellular process, biological regulation and response to stimulus process. After the gene screening, KEGG pathway analysis showed that the target genes are involved in multiple pathways, including the lysine degradation, glycosaminoglycan biosynthesis-chondroitin sulfate/dermatan sulfate and pyruvate metabolism pathways. By combining the results of PPI network and KEGG pathway analyses, we identified seven key node genes. The qRT-PCR results confirmed that the expression of the RHOA, MAPK4 and DLAT genes was significantly down-regulated compared with the levels in DM group, whereas the expression of the SgK494, DOT1L, SETD2 and ME3 genes was significantly up-regulated in the BBR group.

**Conclusion:**

Berberine can significantly improve glucose metabolism and has a protective effects of liver and kidney function in ZDF rats. The qRT-PCR results for the crucial DEGs validated the microarray results. These results suggested that the RHOA, MAPK4, SGK494, DOT1L, SETD2, ME3 and DLAT genes are potential therapeutic target genes for the treatment of diabetes.

## Introduction

Diabetes mellitus (DM), which is characterized by disordered lipid metabolism and a high circulating blood glucose level, is a metabolic syndrome [1]. The main form of diabetes found in 90~95% of diabetic patients globally is type 2 diabetes mellitus (T2DM) [2]. Diabetes can cause many complications, including neuropathy, nephropathy and retinopathy [3]. Diabetes seriously affects the quality of life of patients.

The anti-diabetes activity of Rhizoma Coptidis was documented in the book “Notes of Elite Physicians” written by Hongjing Tao 1500 years ago [3, 4]. Berberine (BBR) is the major isoquinoline alkaloid constituent of the Chinese herb *Rhizoma Coptidis* [1,5,6,7], which has the beneficial characteristic of regulating glucose and lipid metabolism and has been extensively used in the treatment of obesity, diabetes and hypercholesterolemia [3]. Zucker diabetic fatty (ZDF) rats are an animal model of type 2 diabetes mellitus due to a leptin receptor gene mutation. [8]. However, the exact anti-diabetic gene targets of berberine in diabetic model rats have not yet been fully elucidated.

Thus, in this study, we examined the hypoglycemic activity of berberine in ZDF rats and showed that berberine treatment changed the levels of gene expression in the liver to improve the diabetic metabolism. To identify the exact gene targets, we screened the genes, constructed a protein-protein interaction network, and validated the expression levels of the crucially differentially expressed genes (DEGs) that were identified by protein-protein interaction network analysis using qRT-PCR.

## Methods

### Groups and treatment

Six-week-old male ZDF rats (housed two per cage) were obtained from the Animal Center of Zhejiang Chinese Medicine University. To induce diabetes symptoms, ZDF^*fa/fa*^ rats were fed high-sugar/high-fat feed for 7 weeks, whereas ZDF^*fa/+*^ rats were fed the basic feed during this period. Afterward, the ZDF^*fa/fa*^ rats were randomly divided into two groups that had a similar degree of hyperglycemia: the model group (DM) and the BBR group (n = 6 per group). The ZDF^*fa/+*^ rats served as the control (Con) group. The start and end of gavage, the mental and dietary status of animals were observed to monitor animal health in every day during the experimental procedure. The rats in the control and model groups were administered 0.5% gum tragacanth (CAS: 9000-65-1, Shanghai Macklin Biochemical Co., Ltd. Shanghai, China) in normal saline via gavage, whereas the rats in the BBR group were administered berberine (K151212, Xi’an Kai Lai Biological Engineering Co., Ltd. Xi’an, China) at 300 mg/kg dissolved in 0.5% gum tragacanth in normal saline via gavage. All of the animals were housed at 25°C with 50~70% humidity and a 12 h light/12 h dark cycle with free access to regular food and water. Before treatment, the first measurements (0 weeks) of the random plasma glucose, HbA1C, AST, ALT, BUN and CREA levels were taken. The random plasma glucose level was measured at the 1^st^, 3^rd^, 5^th^, 8^th^, 10^th^, and 12^th^ weeks and the HbA1C, AST, ALT, BUN and CREA levels were measured at the 12^th^ week. Throughout the experimental period, no animals died prior to the experimental endpoint. Blood samples were collected from heart after pentobarbital sodium (45mg/kg, i.p.) anesthesia, and then rapidly excised liver tissues, which were quickly frozen in liquid nitrogen, and stored at -80°C for use in the microarray and quantitative real-time PCR assays. At the end of the experiments, euthanasia occurred under sodium pentobarbital anesthesia followed by cardiac puncture/livers removal for all animals. This study was approved by the animal care and welfare committee of Zhejiang Chinese Medical University (permit number: ZSLL-20130-106). All of the procedures involving animals were performed under sodium pentobarbital anesthesia to minimize their suffering.

### Measurement of Blood Biochemical Parameters

#### Random plasma glucose levels in ZDF rats after berberine administration

Sera were prepared from the blood collected at 0 weeks and at weeks 1, 3, 5, 8, 10, and 12 of the treatment via tail vein puncture. The random plasma glucose levels were measured using accu-chek^®^ Performa (Roche Co., Ltd. Shanghai. China).

#### Glycosylated hemoglobin (HbA1C) in ZDF rats after berberine administration

Blood was collected at 0 weeks and at the 12^th^ week of treatment via tail vein puncture. The levels of glycosylated hemoglobin (HbA1C) were determined using a glycosylated hemoglobin analyzer (Quotient Diagnostics Ltd.).

#### AST, ALT, BUN and CREA levels in ZDF rats after berberine administration

Blood samples were obtained by puncturing retro-orbital plexus at 0 weeks and collected from heart under anesthesia at 12^th^ week. The serum samples were obtained by centrifugation (1000g × 10 min, 4°C) for the measurement of blood biochemical parameters. AST, ALT, BUN and CREA levels were measured by a 7020 full automatic biochemical analyzer (Hitachi, Japan).

#### Glucose tolerance test

Upon completion of 12 h fasting, before the start as 0 weeks and end as 12^th^ week of the study. Blood glucose was measured by withdrawing blood from tail vein. All rats received 2.5g/kg glucose by oral gavage, and blood glucose level was measured at four time points (0, 30, 60, 120 min after gavage). Respectively, the area under the curve of oral glucose tolerance test (AUC_OGTT_) was measured through 1/4*(a+b)+1/4*(b+c)+1/2*(c+d), a,b,c,d stand for 0, 30, 60, and 120 min’s blood glucose level. The blood glucose level observed between 30, 60, and 120 min, among various groups compared to 0 min, was used for assessing glucose tolerance. The glucometer used was the accu-chek^®^ Performa (Roche Co., Ltd. Shanghai. China).

### RNA preparation and microarray assay

The liver samples acquired from the control, DM, and BBR groups (n = 3 per group) were utilized in a microarray assay. Agilent rat gene expression 4x44K microarrays, which cover approximately 26,930 genes, were chosen for this study. The total RNA was purified using a QIAGEN RNeasy Mini Kit according to the manufacturer’s instructions (Qiagen, 217004). The quantity of the total RNA in each sample was determined using a NanoDrop instrument, and the RNA integrity was assessed using an Agilent Bioanalyzer 2100 instrument (Agilent Technology, Santa Clara, CA, USA). The microarray assay was independently repeated in triplicate. After hybridization and washing, the microarray chips were scanned using a DNA chip scanner (Agilent Technologies). Feature Extraction software was used to determine the signal intensities.

### GO term enrichment and pathway analysis

The Gene Ontology (GO) platform (http://www.geneontology.org) is a community-based bioinformatics resource in which terms describing gene-product attributes are organized in three structured networks [9]. GO enrichment analysis was performed for the 91 genes with significantly increased expression and the 63 genes with significantly decreased expression using the Gene Ontology database. We defined the significance of GO term enrichment for the DEGs according to the *P*-value, with a cut-off value of 0.05 [9]. We assessed the liver DEGs according to their association with the biological process, molecular function and cellular component categories.

The pathways corresponding to the DEGs were investigated using the KEGG Orthology Based Annotation System 2.0 database (http://kobas.cbi.pku.edu.cn/), which is the most recent version of KOBAS. The function of KOBAS is to identify the human diseases and significantly enriched pathways that correspond to a given set of genes. The significance and selection of the associated pathways was based on the *P*-value, with a cut-off value of 0.05 [9].

### Protein-protein interaction (PPI) network construction

The Search Tool for the Retrieval of Interacting Genes/Proteins (STRING) database (http://string-db.org/) identifies the interactions of gene products, including not only the direct physical interactions of proteins but also their functional interactions. To evaluate the interactions among the DEGs, we uploaded these genes and drew a color-coded protein-protein interaction network graph. We then imported the PPI data in text format into the Cytoscape program (http://www.cytoscape.org/) to visualize the relationships and used its network analyzer plug-in to analyze the PPI network.

### Quantitative real-time PCR analysis

Quantitative real-time PCR (qRT-PCR) analyses were performed using SYBR Green to validate the microarray and PPI network data. Total RNA was isolated from the whole livers of rats (n = 6 per group) using the TaKaRa MiniBEST Universal RNA Extraction kit (TaKaRa, Clontech). The amount and quality of the total RNA were determined using spectrophotometry at 260/280 nm (Thermo Scientific, USA). The RNA was reverse-transcribed using PrimerScript^TM^ RT Master Mix (Perfect Real Time). Previously published primers were used to amplify the RHOA, MAPK4, DOT1L, SETD2, ME3 and DLAT genes [10,11,12,13,14], and primers obtained from PrimerBank (https://pga.mgh.harvard.edu/primerbank/) were used to amplify the SGK494 gene (Table 1). GAPDH was used as the internal control. The RHOA, MAPK4, SGK494, DOT1L, SETD2, ME3 and DLAT amplification conditions were as follows: pre-denaturation at 95°C for 30 sec, followed by 40 cycles of denaturation at 95°C for 5 sec and annealing at 60°C for 30 sec. The dissolution curve conditions were 65°C for 0.05 sec and 95°C for 0.5 sec. The relative mRNA expression levels in the Con, DM and BBR groups were calculated using the comparative Ct method.

**Table 1:** Primers used for Q-PCR.

Gene symbol	Forward primer (5’-3’)	Reverse primer (5’-3’)
RHOA	CATCCCAGAAAAGTGGACTCCA	CCTTGTGTGCTCATCATTCCG
MAPK4	GGACGTCAACAGTGAAGCCATTGA	TCGATCTCGTCCTCAATGCGGAAA
SGK494	GGAGCAGTAAGCTGTCGGC	CCCGTAGTTCCCAGAGTTCTT
DOT1L	AGAATCCGAACGACTCGACAG	CTGTTCTTGGTCTTCGTTCAAC
SETD2	CCTACAGGACATCTGGAGTTAC	GAATCAGTACCAGCATTTAGATG
ME3	TGAAGAAGCGCGGATACGA	GATTAGGCCGTGGATTCCAAG
DLAT	AAAGCCACTGTTGGATTTGAG	TACAGATGATCGCTCCGATG
GAPDH	GATGGGTGTGAACCACGAGAAA	ACGGATACATTGGGGGTAGGAA

RHOA, ras homolog gene family, member A; MAPK4, mitogen-activated protein kinase 4; Sgk494, uncharacterized serine/threonine-protein kinase SgK494; Dot1l, DOT1-like, histone H3 methyltransferase; Setd2, SET-domain containing 2; Me3, malic enzyme 3; DLAT, dihydrolipoamide S-acetyltransferase.

### Statistical analysis

The statistical analysis was performed using SPSS17.0 software. The data presented are the mean values ± standard deviation (SD), with *P<0*.*05* indicating a significant difference. The figures were produced using GraphPad Prism 5 software.

## Results

### Berberine decreased the random plasma glucose level of DM rats

**Fig 1:**
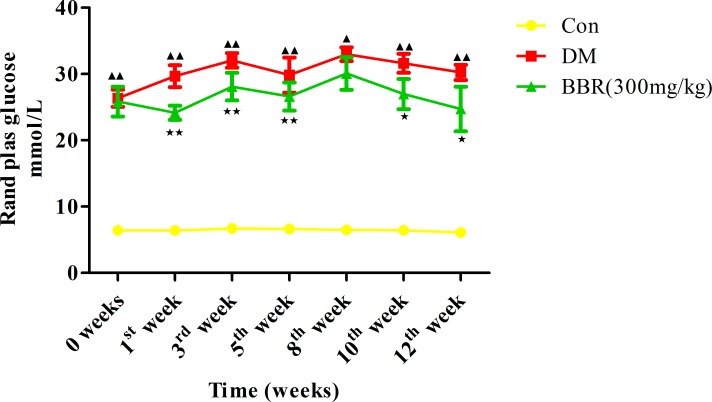
Effect of daily oral administration of berberine for 12 consecutive weeks on the random plasma glucose level. ^▲^*P<0*.*05*,^▲▲^*P<0*.*01* the DM group versus the control group; ^★^*P<0*.*05*, ^★★^*P<0*.*01* the BBR group versus the DM group. The data presented are the mean values ± SD, *n* = 6.

The random plasma glucose level of the DM rats was significantly higher than that of the control rats at 0 weeks (*P<0*.*01*) and at the 1^st^ week (*P<0*.*01*), 3^rd^ week (*P<0*.*01*), 5^th^ week (*P<0*.*01*), 8^th^ week (*P<0*.*01*), 10^th^ week (*P<0*.*01*), and 12^th^ week of the experimental period (*P<0*.*01*). The random plasma glucose level in the BBR group was significantly lower at the 1^st^ week (*P<0*.*01*), 3^rd^ week (*P<0*.*01*), 5^th^ week (*P<0*.*01*), 10^th^ week (*P<0*.*05*), and 12^th^ week (*P<0*.*05*) of treatment than the levels in the DM group (Fig 1).

### Berberine reduced the glycosylated hemoglobin (HbA1C) level of DM rats

**Fig 2:**
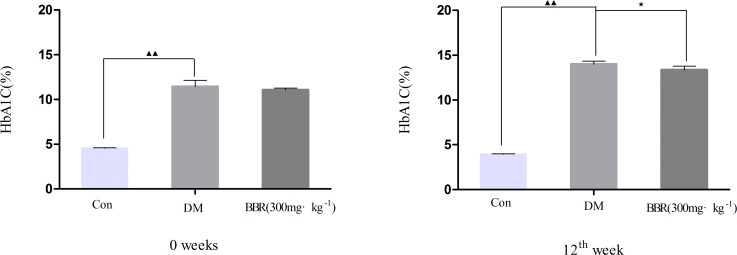
Effect of daily oral administration of berberine for 12 consecutive weeks on the level of glycosylated hemoglobin (HbA1C). ^▲▲^*P<0*.*01* versus the control group; ^★^*P<0*.*05*, ^★★^*P<0*.*01* versus the DM group. The data presented are the mean values ± SD, number of rats per group *n* = 6.

At 0 weeks and at the 12^th^ week of the experimental period, the HbA1C levels were higher in the DM group than in the control group (*P<0*.*01*) (Fig 2). The HbA1C level of the DM rats treated with BBR for 12 weeks was significantly reduced compared with that of the untreated DM rats (*P<0*.*05*) [6].

### Effect on AST, ALT, BUN and CREA

**Fig 3:**
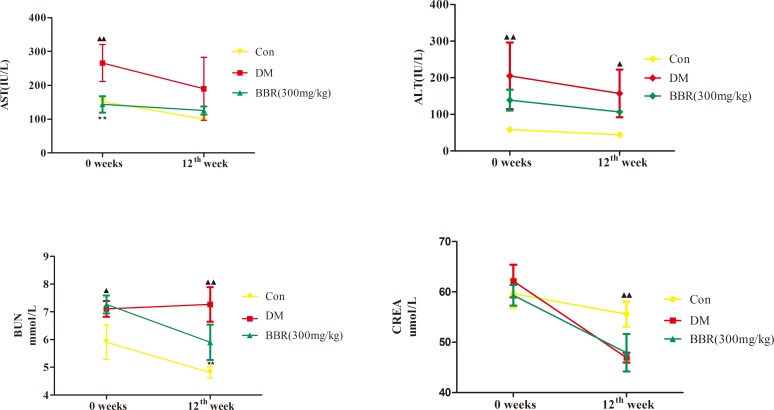
Effect of daily oral administration of berberine for 12 consecutive weeks on the serum levels of AST, ALT, BUN and CREA in ZDF rats. ^▲^*P<0*.*05*, ^▲▲^*P<0*.*01* the DM group versus the control group; ^★★^*P<0*.*01* the BBR group versus the DM group. Data are expressed as mean ± SD, number of rats per group *n* = 6. AST: Aspartate aminotransferase; ALT: alanine aminotransferase; BUN: blood urea nitrogen; CREA: creatinine.

The biochemical results showed that the DM group with a significant increase (*P<0*.*01 or P<0*.*05*) in the serum marker enzymes aspartate, alanine transaminases and blood urea nitrogen (AST, ALT and BUN) at 0 weeks. Under our experimental conditions, there were no statistical differences in AST, ALT and CREA between the BBR group and DM group (*P>0*.*05*), but expect the BUN at the 12^th^ week (*P<0*.*01*; Fig 3).

### Effect on glucose tolerance

As shown in Table 2, the blood glucose level and the area under the curve (AUC) were significantly elevated in the DM group when compared with the normal control group (*P<0*.*01*) at 0 weeks and at the 12^th^ week. After 12 weeks of berberine treatment, the area under the curve (AUC) level was significantly reduced in BBR group (*P<0*.*05*) compared with DM group.

**Table 2:** Effect of berberine treatment on glucose tolerance test in ZDF rats.

0 weeks	Groups	Time/min				AUC/mmol ⋅L^−1^⋅h^−1^
		0	30	60	120	
	Con	5.97±0.48	7.90±0.32	7.52±0.41	5.67±0.35	13.91±0.47
	DM	17.25±4.06^▲▲^	28.42±2.83^▲▲^	29.60±2.04^▲▲^	25.83±3.29^▲▲^	53.64±5.19^▲▲^
	BBR	14.23±5.51	24.67±5.27	28.22±6.77	22.80±6.72	48.46±12.21
12^th^ week	Groups	Time/min				AUC/mmol ⋅L^−1^⋅h^−1^
		0	30	60	120	
	Con	5.90±0.73	6.83±1.04	5.57±1.17	4.80±0.90	11.47±1.58
	DM	27.68±1.18^▲▲^	33.40±0.00^▲▲^	33.35±0.12^▲▲^	29.27±0.64^▲▲^	63.27±0.52^▲▲^
	BBR	25.08±1.07^★★^	33.37±0.08	32.92±1.14	28.67±0.88	61.98±1.02^★^

AUC, area under curve. Data presented as the mean values ± standard deviation (SD).

^▲▲^*P<0*.*01* compared to normal control (Con)

^★★^*P<0*.*01*

^★^*P<0*.*05* compared to diabetic control (DM).

### Microarray analysis of gene expression

**Fig 4:**
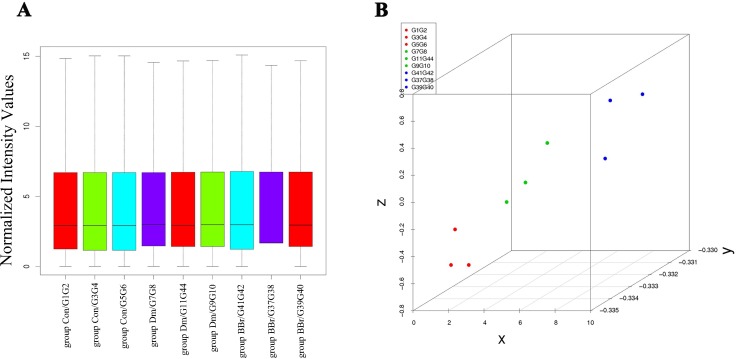
Plots of all of the differentially expressed genes. A. Box plot of the genes that were differentially expressed in the Con, DM and BBR groups; B. PCA plot of the genes that were differentially expressed in the Con, DM and BBR groups. The plot was constructed based on the fold-change in expression level and the significance of the differences, as determined from the *P*-values.

The gene expression data obtained using the Agilent rat gene expression 4x44K microarray were extracted using GenePix pro 6.1 software (Agilent Technologies). Quantile normalization and subsequent data processing were performed using the Agilent_Analyze_V1.0 program (Agilent Technologies). Quantile normalization and PCA analysis of the raw data (Fig 4A and Fig 4B) showed that our data were symmetrical and representative. Among the DEGs (S1 Table) detected using microarray analysis, 661 genes had differentially up-regulated expression and 546 genes had differentially down-regulated expression (S2 Table). The genes with log2 (Con vs DM) ≥ 1 and log2 (BBR vs DM) ≥ 1 expression levels and the genes with log2 (Con vs DM) ≤ -1 and log2 (BBR vs DM) ≤ -1 expression levels at *P*<0.05 were combined, leading to the identification of 154 genes with significantly different expression levels in the livers of the BBR group. Of these genes, 91 had significantly increased expression levels and 63 had significantly decreased expression levels (S3 Table).

### GO enrichment and pathway analysis

**Fig 5:**
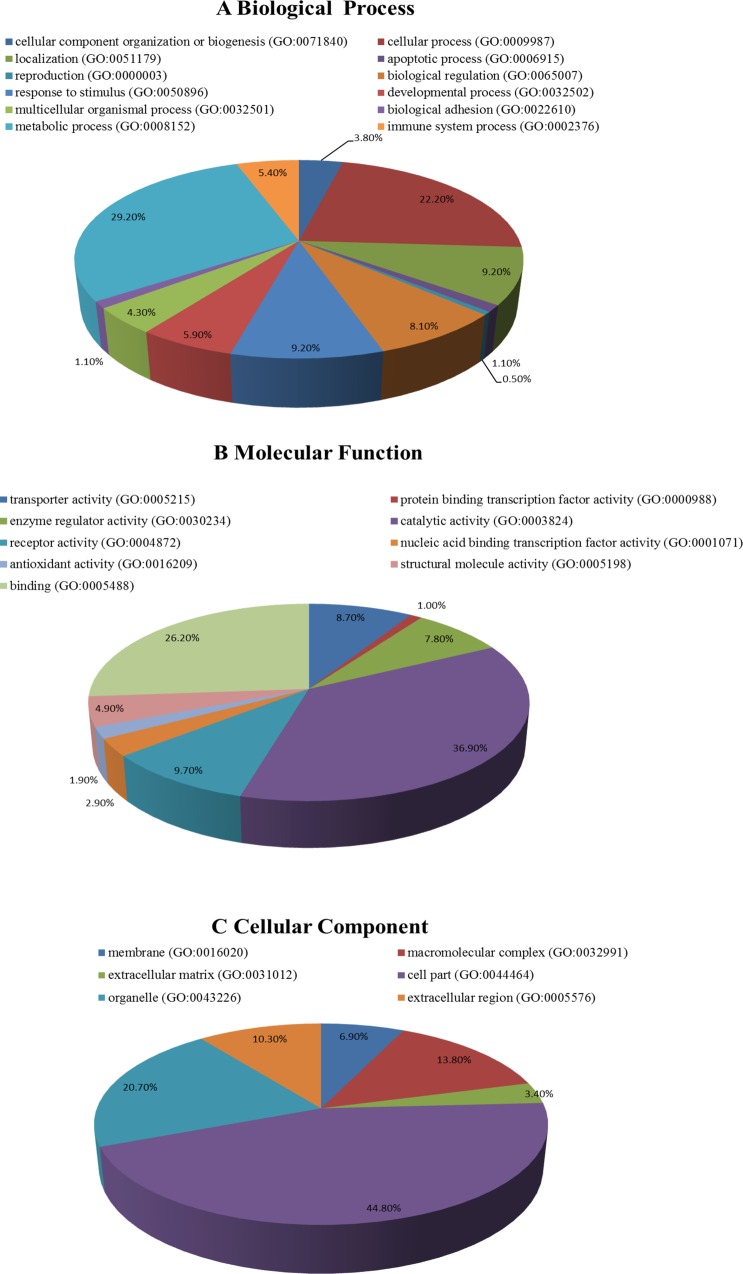
GO enrichment analysis of the differentially expressed genes detected in the liver samples. (A) Biological process enrichment; (B) molecular function enrichment; (C) cell component enrichment.

Both up-regulated and down-regulated genes were observed in the liver samples of the ZDF rats. GO analysis revealed that the functions of these genes are related to many biological processes that are important for the occurrence and development of diabetes, such as the processes in the cellular component organization or biogenesis, metabolic process, biological regulation and response to stimulus categories. The molecular functions associated with these genes include transporter activity, catalytic activity, enzyme regulator activity and antioxidant activity, and the cellular components corresponding to these genes include organelle, cell part, macromolecular complex and extracellular region (Fig 5).

The main pathways for both the up-regulated genes and the down-regulated genes are the lysine degradation, glycosaminoglycan biosynthesis-chondroitin sulfate/dermatan sulfate, thyroid hormone synthesis, hematopoietic cell lineage, and pyruvate metabolism pathways (Table 3).

**Table 3:** KEGG pathway enrichment results for the genes with either up-regulated or down-regulated expression.

Pathway name	Database	ID	Gene symbols	*P*-value
Lysine degradation	KEGG PATHWAY	rno00310	Dot1l, Setd2	0.0088
Glycosaminoglycan biosynthesis—chondroitin sulfate/dermatan sulfate	KEGG PATHWAY	rno00532	Dse, Chst13	0.0135
Thyroid hormone synthesis	KEGG PATHWAY	rno04918	Atf6b, Asgr1, Gpx3	0.0183
Hematopoietic cell lineage	KEGG PATHWAY	rno04640	Tfrc, Cd8b	0.0283
Pyruvate metabolism	KEGG PATHWAY	rno00620	DLAT, Me3	0.0418

KEGG, Kyoto Encyclopedia of Genes and Genomes; Dot1l, DOT1-like, histone H3 methyltransferase; Setd2, SET-domain containing 2; Dse, dermatan sulfate epimerase; Chst13, carbohydrate (chondroitin 4) sulfotransferase 13; Atf6b, activating transcription factor 6 beta; Asgr1, asialoglycoprotein receptor 1; Gpx3, glutathione peroxidase 3; Tfrc, transferrin receptor; Cd8b, CD8b molecule; DLAT, dihydrolipoamide S-acetyltransferase; Me3, malic enzyme 3, NADP(+)-dependent, mitochondria.

### Interaction network construction and network analysis

**Fig 6:**
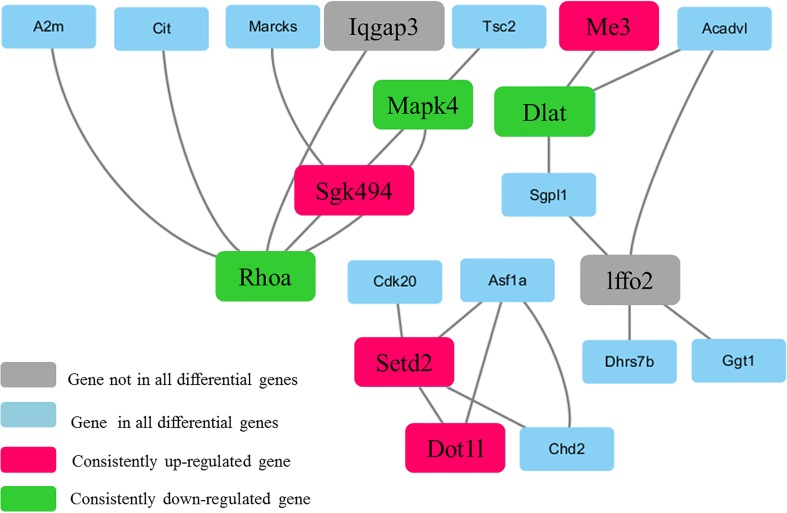
The module identified in the protein-protein interaction network constructed using Cytoscape software. Proteins encoded by up-regulated (red) and down-regulated (green) genes are indicated by color.

We evaluated 154 DEGs using the STRING version 10.0 database to identify the interactions among their gene products; this analysis identified 145 proteins. Then, a PPI network was constructed using Cytoscape software (Fig 6) to provide insight into the molecular mechanisms underlying the actions of the DEGs in the liver However, the network contained many isolated nodes, which did not provide useful information [15]. Therefore, we deleted the DEGs corresponding to isolated nodes and pairs of linked nodes, including Ppm1b, Atp2c1, Sod3, Lyc2, Hrh4, Drd2, Rpl3, RGD1565713, Rps2, Gar1, Hnrnpa3, Clk1, and Srsf7, among others (S3 Table). The resulting network was composed of 20 nodes and 21 edges, with 5 as the maximum degree of connectivity of a node and 1 as the minimum. The average degree of connectivity of the nodes in the network was 2.1. When the degree of connectivity of a node was much greater than average, the node was considered significantly more important than the other nodes and was called the central node. The central node of a PPI network is the core protein in the network. In this study, the nodes were analyzed using a network analyzer, with the degree of connectivity of a node set as ≥ the mean value for all nodes to identify candidate therapeutic target genes. RHOA, MAPK4, Sgk494, Setd2 and DLAT were identified as such genes. Based on the results of the KEGG pathway and protein-protein network analyses, Dot1l and Me3 were also identified as candidate target genes.

**Fig 7:**
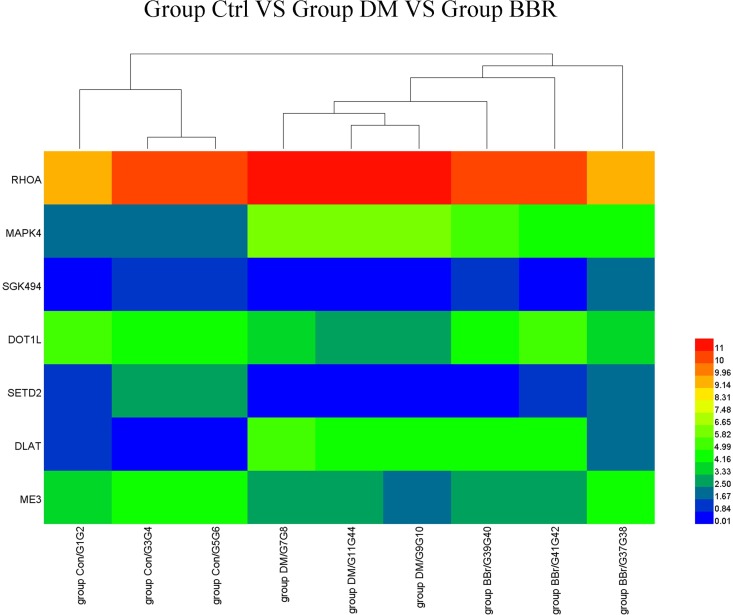
Heat map of the DEG expression levels. Heat map showing the expression levels of the seven genes that were differentially expressed in the liver samples taken from the Con (n = 3), DM (n = 3) and BBR (n = 3) groups. Each row represents one gene, and each column represents a liver sample. The gene symbols are shown on the left side of the rows. The relative gene expression levels are depicted according to the color scale shown at the right. Red indicates high-level expression; blue indicates low-level expression.

The seven genes with significantly different expression levels in nine liver samples (three each from the Con, DM, and BBR groups) were used to generate a heat map based on hierarchical clustering analysis of the normalized microarray expression data. The liver samples were collected from randomly selected members of the control, DM, and BBR groups (Fig 7).

### Validation of the microarray data using real-time qRT-PCR

**Fig 8:**
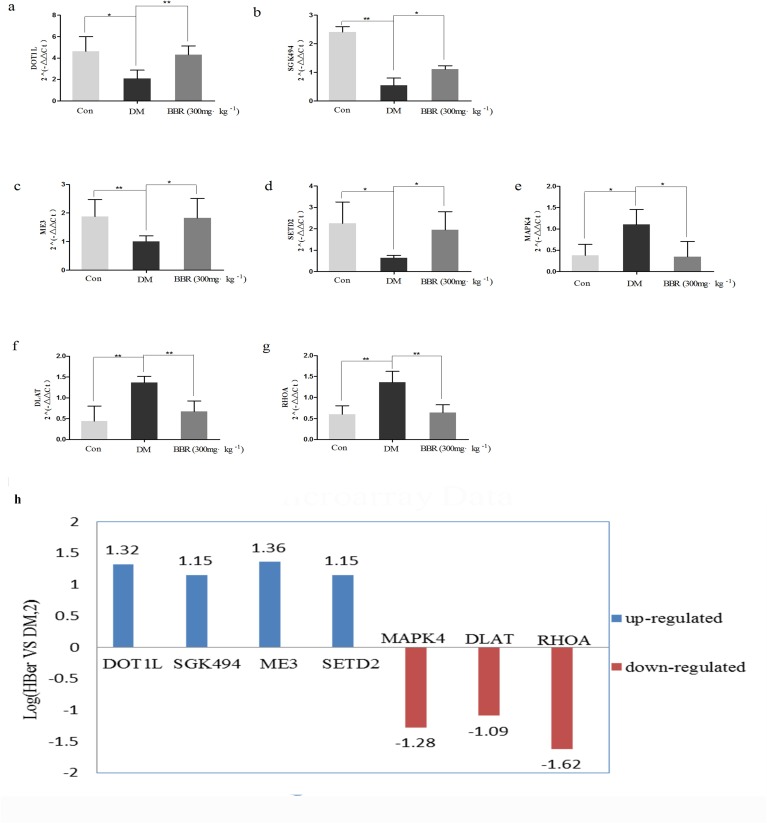
Validation of the microarray data using qRT-PCR. Relative expression levels of the seven DEGs (RHOA, MAPK4, SGK494, DOT1L, SETD2, ME3, and DLAT) determined using qRT-PCR. The *P*-values were calculated using a one-way ANOVA (**P<0.01;*P<0.05). The qRT-PCR results were consistent with the microarray data.

The validity of the microarray-based identification of genes with significantly different expression in the liver was confirmed using qRT-PCR. This method identified seven genes with significantly different expression. As shown in Fig 5, the expression levels of DOT1L, SGK494, ME3 and SETD2 were significantly higher in the BBR group than in the DM group (Fig 8A, Fig 8B, Fig 8C and Fig 8D), whereas the expression levels of MAPK4, DLAT and RHOA were significantly lower in the BBR group than in the DM group (Fig 8E, Fig 8F and Fig 8G). The expression level of each of these seven DEGs was significantly different at *P<0*.*05*, demonstrating that the qRT-PCR results regarding the seven DEGs were consistent with the microarray results (Fig 8H).

## Discussion

The results of our study showed that berberine treatment of ZDF rats, which exhibit significant hyperglycemia as well as insulin resistance, obesity and hyperlipidemia, significantly reduced their random plasma glucose and HbA1C levels. These results suggest that berberine exerts its anti-diabetic effect in ZDF rats by moderating the diabetic metabolism. These results are similar to those obtained in previous studies [7,16,17,18,19]. The administered dosage (300mg/kg) of berberine was determined according to the equation used for the adult maximal dosage in the clinic [7].The toxicity effect of berberine was evaluated by assaying liver and kidney function markers, the aspartate aminotransferase (AST), alanine aminotransferase (ALT), blood urea nitrogen (BUN), creatinine (CREA). Although the biochemical results showed no marked statistical differences, but we still observed a tendency, the hepatoprotective and renoprotective effect of berberine by regulating liver and kidney function markers under our experimental conditions. The pathogenesis and pathological features of diabetes are not yet clear, and the vast majority of researchers consider diabetes to be a multifactorial disease. GO analysis of the genes with differentially down-regulated and up-regulated expression in the BBR group indicated that these genes were associated with the categories of metabolic process, cellular process (ontology: biological process), catalytic activity, binding (ontology: molecular functions), cell part, and organelle (ontology: cellular functions). Interestingly, in studying the long noncoding RNAs that were differentially expressed in sperm samples collected from diabetic and non-diabetic mice, Jiang [9] found that these RNAs were associated with the GO categories of metabolic process, cell part, binding and catalytic activity. The results of the present study are consistent with these findings. KEGG pathway analysis revealed that some of the genes that were differentially expressed in the BBR group are involved in lysine degradation and pyruvate metabolism. Lysine acetylation is vital for metabolic pathway activity and may regulate the balance between energy storage and expenditure [20]. Pyruvate is a central biochemical node connecting amino acid, carbohydrate, and fatty acid metabolism [21]. We found that the expression levels of DOT1L and SETD2, which are involved in lysine degradation, were up-regulated in the BBR group. In addition, the expression levels of two genes involved in pyruvate metabolism were significantly altered in the BBR group, with down-regulation of DLAT and up-regulation of ME3. Lysine and pyruvate both play important roles in glycometabolism. Consequently, the results of our study provide a basis for research on the pathogenic mechanisms of diabetes at the molecular level.

The results of the PPI network and pathway analyses of the genes that were significantly differentially expressed in the livers of the ZDF rats suggest that seven genes, RHOA, MAPK4, SGK494, DOT1L, SETD2, ME3 and DLAT, play important roles in diabetes and are candidate targets for diabetes treatment.

Ras homolog gene family, member A (RHOA) is an important member of the Rho small G protein superfamily, which regulates cytoskeletal architecture and interacts with the downstream critical target ROCK to affect cell shrinkage, proliferation, migration and adhesion. The RHOA/ROCK signaling pathway plays critical roles in the heart, liver, lungs and kidneys [22]. Excessive activation of the RHOA/ROCK signaling pathway plays an important role in the development of the chronic complications of diabetes [23]. RHOA also involved in renal integrity in diabetic mice, and its inhibitors attenuate diabetic nephropathy in different models of diabetes [24]. RHOA/ROCK signaling activated by TGF-β to regulate Nox4 activity, which might be associated with abnormalities in renal structure [25]. Berberine ameliorates the microvascular complications of diabetes, the renoprotective and anti-oxidative stress effect of BBR by inhibiting the activation of RHOA/ROCK signaling [26]. RHOA was significantly down-regulated in the BBR group in this study, which is consistent with these findings.

Mitogen-activated protein kinase 4 (MAPK4) is a protein serine/threonine kinase that specifically regulates the activity of mitogen-activated protein kinase. The MAPK-mediated signaling pathway plays a major role in cell growth, proliferation, differentiation and apoptosis. In addition, this pathway is involved in insulin resistance and protein phosphorylation. Up-regulated MAPK4 expression can lead to insulin resistance, and MAPK4 overexpression inhibits insulin-stimulated glucose uptake by negatively regulating the insulin signal-transduction pathway [27]. The ZDF rat diabetic model used in this study is a typical model of insulin resistance. The microarray and PCR results showed that the MAPK4 expression was significantly increased in the DM group compared with the control group. In contrast, the MAPK4 expression was significantly decreased in the BBR group compared with the DM group.

Uncharacterized serine/threonine-protein kinase (SGK494) is an important member of the AGC protein kinase family. AGC kinases not only are involved in diverse cellular functions but also may be associated with human diseases, such as diabetes, obesity, inflammation and viral infections [28,29]. To the best of our knowledge, no studies further elucidating the role of this kinase in diabetes have been conducted, and there are no reports of the effect of BBR treatment on SGK494. Our study is the first to show the increased expression of SGK494 in BBR-treated ZDF rats.

DOT1L, a nucleosomal histone 3 (H3)-specific methyltransferase, participates in a variety of cellular processes, including reprogramming, development, differentiation, and proliferation [30]. Zhou et al. found that DOT1L encodes histone H3K79 methyltransferase Dot1a, which contributes to the recovery of renal injury in streptozotocin-induced diabetic rats [31].DOT1L deficiency results in the upregulation of endothelin-1 (ET-1) expression at both mRNA and protein levels in diabetic kidney, and suggest that associated with decreased expression of Dot1a [31]. DOT1L also regulates water homeostasis, and identify AQP5 as a potential novel target of Dot1a, an AQP2 binging partner and regulator. Moreover, depletion of DOT1L led to endogenous AQP5 upregulation, but the upregulated AQP5 contribute to polyuria in patients with diabetic nephropathy, suggesting that DOT1L is critical in renal water homeostasis with DN [32]. In the current study, the PCR results showed higher expression of DOT1L in the BBR group than in the DM group, suggesting that the DOT1L is a potential target about the renoprotective effect of BBR.

SET domain-containing 2 (SETD2) is a histone methyltransferase that trimethylates lysine 36 of H3 (H3K36) and is involved in transcriptional elongation, RNA processing, and DNA repair [33]. The H3K36 site can be modified by methylation, and its modified state is controlled by the dynamic regulation of the methyl transferase and the demethylase that are specific to H3K36. Histone demethylation plays a role in the transcriptional regulation of gluconeogenesis, which has important implications for the treatment of diabetes [34].

Malic enzyme (ME) is a lipogenic enzyme whose activity level is correlated with the rate of fatty acid synthesis [13]. The mitochondrial NADP malic enzyme (ME3) is one isoform of ME. The function of mitochondrial MEs is to supply adequate amounts of pyruvate for the operation of the Krebs cycle [35], and their expression is synergistically regulated by insulin and glucose [36]. Hasan et al. found that ME3 participates in the insulin secretion by pancreatic β-cells, and knockdown of ME3 inhibited insulin release stimulated by glucose, pyruvate or 2-aminobicyclo [2,2,1] heptane-2-carboxylic acid-plus-glutamine, suggesting that ME3 far more than ME1 or ME2 is necessary for insulin release [37]. The knockdown of ME3 led to the inhibition of insulin release might cause by lowering its malic enzyme catalytic activity. In this study, we found that ME3 was expressed at a higher level in the BBR group than in the DM group might suggest that BBR improves insulin release through increase the expression of ME3 and its catalytic activity.

Dihydrolipoamide S-acetyltransferase (DLAT) is the E2 component of the large mitochondrial pyruvate dehydrogenase-containing complex that catalyzes the conversion of pyruvate to acetyl coenzyme A [38], and a responsible gene for pyruvate dehydrogenase complex defect causes of mitochondrial disorders [39]. Moreover, Goguet-Rubio et al revel a central role for DLAT in the pyruvate metabolism program regulated through E4 transcription factor 1 [40]. Lee et al. used cDNA microarray analysis to study the placental gene expression related to glucose metabolism and found nine genes with altered expression levels, including DLAT, which related to the tricarboxylic acid cycle pathway. Through increased mRNA and protein expression of DLAT to increase glycolysis and gluconeogenesis [41]. In this study, we found that the DLAT expression was significantly down-regulated in the BBR-treated ZDF rats. Exact mechanisms whereby BBR and DLAT interact and how this impacts on pyruvate metabolism warrant further investigation.

In this study, we did not focus on the DEGs represented as pairs of linked nodes in the PPI network. However, these genes may play roles in diabetes; one of these genes encodes Ppm1b (protein phosphatase, Mg^2+^/Mn^2+^ dependent, 1B, Ppm1b), which increases the expression of PPARγ. In adipocytes, PPARγ not only promotes the removal of lipids and free fatty acids (FFAs) but also inhibits the production of proinflammatory cytokines, ameliorating insulin resistance [42]; this finding suggests that Ppm1b and other genes may be worth further study under certain conditions. Therefore, we will investigate the simply linked genes in a follow-up study.

In conclusion, based on current experimental evidence, our study showed that BBR treatment improved the glucose metabolism and has protective effects against hepatorenal impairment of diabetic rats. We found that the RHOA, MAPK4, SGK494, DOT1L, SETD2, ME3 and DLAT genes were significantly differentially expressed in the livers of the BBR and DM groups and thus are potential targets for the treatment of T2DM. These results provide molecular information that will facilitate elucidating the mechanisms through which berberine alleviates T2DM, and further studies are required to confirm the results of this study.

## Supporting Information

S1 Table The differentially expressed genes identified in the control, DM and BBR rat liver samples. (XLSX)

S2 Table The 661 up-regulated and 546 down-regulated genes identified in the control, DM and BBR rat liver samples. (XLSX)

S3 Table The 91 up-regulated and 63 down-regulated genes identified in the control, DM and BBR rat liver samples. (XLSX)
